# Clinical Presentation and Long-Term Outcomes of Lupus Nephritis in Palestine: A Multicenter Retrospective Study

**DOI:** 10.7759/cureus.92561

**Published:** 2025-09-17

**Authors:** Mohammed AR Abdellatif, Ahmed Qaddoumi, Eyas Jawabreh, Mohammad Kayed, Ahmad Zohud, Mohanad Hassan, Mohammad Bourini, Abdullah Hamamdeh, Ahmad Enaya, Hanood Abu Rass

**Affiliations:** 1 Department of Medicine, Palestine Medical Complex, Ramallah, PSE; 2 Department of Medicine, An-Najah National University, Nablus, PSE; 3 Department of Nephrology, An-Najah National University Hospital, Nablus, PSE; 4 Department of Nephrology, Al-Makassed Hospital, Jerusalem, PSE; 5 Department of Internal Medicine, Al-Makassed Hospital, Jerusalem, PSE; 6 Department of Internal Medicine, An-Najah National University Hospital, Nablus, PSE; 7 Department of Pathology, An-Najah National University Hospital, Nablus, PSE

**Keywords:** hematuria, hypertension, lupus nephritis, proteinuria, systemic lupus erythematosus

## Abstract

Introduction: Systemic lupus erythematosus (SLE) is a systemic autoimmune disease that frequently affects the kidneys, resulting in lupus nephritis (LN). LN significantly impacts patient morbidity and mortality, with varying clinical presentations and treatment responses across populations. This retrospective study aimed to describe the demographic, clinical, laboratory, and histopathological features of Palestinian patients with biopsy-confirmed LN, and to assess treatment strategies and patient outcomes.

Methods: This multicenter retrospective study was conducted between November 2023 and May 2024 across three major Palestinian hospitals: An-Najah National University Hospital, Al-Watani Hospital, and Al-Makassed Hospital. We reviewed the medical records of patients with SLE who underwent renal biopsy between July 2016 and December 2023. A total of 78 patients with SLE who had biopsy-confirmed LN were included. For each patient, demographic characteristics (age, sex, family history), clinical features (comorbidities, systemic manifestations), and laboratory parameters (serum creatinine, blood urea nitrogen (BUN), proteinuria, hematuria, complement levels, antinuclear antibody, and anti-double-stranded DNA) were extracted from medical records. Histopathological findings were reviewed using light microscopy and immunofluorescence. Treatment modalities, including corticosteroids, disease-modifying antirheumatic drugs (DMARDs), and additional immunosuppressive agents, were documented. Outcomes were categorized into remission, progression to end-stage renal disease (ESRD), or death. Statistical analyses were performed using IBM SPSS version 22 (IBM Corp., Armonk, NY), with descriptive statistics applied to summarize patient characteristics and Fisher’s exact test employed to examine associations between variables and outcomes.

Results: A total of 78 patients with biopsy-confirmed LN were analyzed, of whom 63 (80.8%) were females. The most common age group was 15-29 years (46.2%). Hypertension (30.7%) and diabetes mellitus (8.0%) were the leading comorbidities. Proteinuria was present in 90.8% of patients, with nephrotic-range proteinuria (>3.5 g/day) in 48.7%, while hematuria was observed in 70.1%. Hypocomplementemia was common, with low C3 in 61.3% and low C4 in 62.1%. The most frequent biopsy classes were class IV (47.1%) and class III (22.9%). Regarding outcomes, 71 patients (91.0%) remained in remission with ongoing treatment, five patients (6.4%) progressed to ESRD requiring dialysis, and two patients (2.6%) died due to LN complications. Male sex was significantly associated with dialysis dependency (p < 0.001), while hypertension was also strongly linked to dialysis outcomes (p = 0.035). Elevated serum creatinine (>1.5 mg/dL) and BUN (>20 mg/dL) were strongly associated with both ESRD and mortality (p < 0.001 and p = 0.01, respectively).

Conclusion: This study sheds light on LN patients in Palestine, highlighting the high morbidity and mortality rates. It emphasizes early detection and aggressive management, especially in patients with hypertension and elevated creatinine levels, to improve outcomes and reduce progression to ESRD.

## Introduction

Systemic lupus erythematosus (SLE) is a chronic autoimmune disease that can affect multiple organs, with the kidneys being among the most commonly involved, which is termed lupus nephritis (LN) [1]. LN affects about 30-60% of adults and around 70% of children diagnosed with SLE [2,3]. It occurs due to the deposition of immunoglobulin and complement in the glomerulus [4]. According to the World Health Organization (WHO) and the International Society of Nephrology/Renal Pathology Society's recommendations, LN is classified into six classes based on the morphologic changes of glomeruli seen under microscopy, immune deposits seen on immunofluorescence, and electron microscopy [5].

Clinical features of LN range from asymptomatic urinary abnormalities to nephrotic syndrome, acute nephritic syndrome, or rapidly progressive glomerulonephritis. Common symptoms include peripheral edema, hypertension, foamy urine, and other systemic SLE manifestations such as malar rash, arthritis, and mucosal ulcers [5,6].

Most SLE patients develop LN within five years of the first SLE symptom. Consequently, routine renal monitoring is recommended from the time of SLE diagnosis, even in asymptomatic patients [1,5]. Investigations that are done include urinalysis that will show proteinuria, hematuria, urinary red blood cell casts, and other urinary cellular casts, serum creatinine concentration or estimated glomerular filtration rate, anti-double-stranded DNA (anti-dsDNA) antibodies, and complement components C3 and C4 [1,7]. However, the gold standard method for LN diagnosis is renal biopsy [7].

The treatment goal for LN is to preserve renal function, achieve clinical and immunological remission, and prevent progression to chronic kidney disease (CKD) and end-stage renal disease (ESRD) [8]. According to recent American College of Rheumatology (ACR)/European League Against Rheumatism (EULAR) recommendations, treatment of LN includes glucocorticoids in combination with either mycophenolate mofetil (MMF) or low-dose intravenous cyclophosphamide as induction therapy. In selected patients, combination regimens such as MMF with belimumab or with calcineurin inhibitors (e.g., voclosporin and tacrolimus) are recommended. Maintenance therapy generally involves MMF or azathioprine, with adjunct biologic agents considered for refractory cases [8]. The five-year survival rate has improved to 80% with this approach, but about 30% of patients still progress to ESRD [1].

In 2011, research was conducted on the prevalence of different classes of LN at Jordan University Hospital from 2002 to 2010. The study was made of 36 renal biopsies, which detected that none of the biopsies were classified as class VI, and the most prevalent class was class IV (63.8%) [9].

In 2019, a retrospective study on 79 patients with LN at a Jordanian tertiary medical center found that 59.5% presented with asymptomatic proteinuria and hematuria. Only 40.5% of biopsy-proven LN cases had anti-DNA positivity. Additionally, 25% of cases developed ESRD, with 80% having class IV LN [10]. In 2021, a study found that the overall incidence of renal failure within 20 years of SLE diagnosis was 8.4% [11]. Proteinuria within the first year of SLE diagnosis was a significant predictor of ESRD, along with African-American ethnicity [11]. This study aims to provide a comprehensive analysis of LN in the Palestinian context by (1) describing patient demographics, clinical and laboratory characteristics, and histopathological classifications; (2) evaluating treatment strategies and responses; and (3) identifying outcomes and prognostic indicators. This focused objective will help guide management and contribute to improving care for this population.

## Materials and methods

This retrospective study was conducted across three major hospitals in Palestine: An-Najah National University Hospital (NNUH), Al-Watani Hospital, and Al-Makassed Hospital. Medical records of patients diagnosed with SLE who underwent renal biopsy between July 2016 and December 2023 were reviewed.

Inclusion criteria were as follows: (1) patients fulfilling the revised ACR criteria for SLE, and (2) those with renal biopsy-confirmed LN, classified according to the 2003 International Society of Nephrology/Renal Pathology Society (ISN/RPS) classification system [5].

Patients were excluded if medical records were incomplete for key variables (clinical, laboratory, or biopsy findings) or if renal biopsy was performed for indications other than LN.

A total of 78 eligible patients were included in the analysis. Renal biopsies were uniformly processed at institutional pathology laboratories following standard protocols. All samples underwent staining with hematoxylin and eosin, periodic acid-Schiff, and Masson’s trichrome, as well as immunofluorescence for IgG, IgM, IgA, C3, C1q, kappa, and lambda light chains. Although electron microscopy was unavailable, classification adhered strictly to ISN/RPS 2003 criteria [5].

For each patient, data were collected on demographics (age and gender), comorbidities (hypertension, diabetes mellitus, cardiovascular disease), and clinical manifestations at the time of renal biopsy. The duration of SLE was defined as the time between the diagnosis of SLE and the biopsy-confirmed diagnosis of LN. Laboratory results at presentation were recorded, including serum creatinine, blood urea nitrogen (BUN), urine protein-to-creatinine ratio, presence of hematuria, serum albumin, hemoglobin, platelet count, antinuclear antibody (ANA), anti-dsDNA, complement levels (C3, C4), erythrocyte sedimentation rate (ESR), and C-reactive protein (CRP). Information on medications used before and after the diagnosis of LN, including corticosteroids, disease-modifying antirheumatic drugs (DMARDs), and other immunosuppressive agents, was also documented.

Patient outcomes were categorized into three groups: (1) those in complete or partial remission who remained under follow-up with ongoing treatment; (2) those who developed ESRD requiring dialysis, and (3) those who died from any cause during follow-up due to complications related to LN.

Data were analyzed using IBM SPSS version 22 (IBM Corp., Armonk, NY). Descriptive statistics were presented as frequencies and percentages for categorical variables, and as means with standard deviations or medians for continuous variables, depending on the distribution of the data. Fisher’s exact test was used to assess associations between categorical variables and outcomes, given the relatively small sample size. A p-value of less than 0.05 was considered statistically significant. Given the retrospective design, potential confounding factors (such as variable treatment exposures and comorbidities) were minimized by stratified analyses, and only biopsy-confirmed cases were included to improve reliability.

The study received ethical approval from the Institutional Review Board (IRB) of An-Najah National University (Reference number: Med. Nov 2023/3). All patient data were anonymized and handled confidentially in accordance with the ethical standards of research.

## Results

A total of 78 patients with biopsy-confirmed LN were included in this study. The majority were female (80.8%), with most patients (46.2%) aged between 15 and 29 years at the time of diagnosis. Regarding comorbidities, 30.7% had hypertension and 8.0% had diabetes mellitus. Most patients (46.2%) were diagnosed with SLE between five and 10 years before undergoing renal biopsy. A positive family history of SLE was rare (1.7%), while 10.0% had a family history of autoimmune diseases, and 9.5% had a history of rheumatologic disorders. Renal biopsy revealed that the most common LN classes were class IV (47.1%) and class III (22.9%). Class V was present in 14.3% of patients, while other classes were less frequently encountered. Notably, 40% of biopsies showed evidence of fibrosis under light microscopy (Table 1).

**Table 1:** Baseline characteristics of the patients.

Variable	Frequency, n (%)
Age at diagnosis	
1-14 years	2 (2.6%)
15-29 years	36 (46.2%)
30-45 years	31 (39.7%)
>45 years	9 (11.5%)
Gender	
Female	63 (80.8%)
Male	15 (19.2%)
History of diabetes mellitus	6 (8%)
History of hypertension	23 (30.7%)
Duration of systemic lupus erythematosus (SLE)	
<1 year	5 (7.7%)
1-4 years	22 (33.8%)
5-10 years	30 (46.2%)
>10 years	8 (12.3%)
Family history of SLE	1 (1.7%)
Family history of Autoimmune disease	6 (10%)
Lupus nephritis class	
Class I	2 (2.9%)
Class II	6 (8.6%)
Class III	16 (22.9%)
Class IV	33 (47.1%)
Class V	10 (14.3%)
Class VI	1 (1.4%)
Class IV+V	2 (2.9%)
Fibrosis (under light microscopy)	31 (40%)

In terms of clinical manifestations, non-erosive arthritis was the most frequently reported symptom, affecting 63.5% of patients. Malar rash was present in 32.5%, and other types of rash were reported in 24.3% of cases. Fever occurred in 14.9% of cases, photosensitivity in 12.8%, and serositis in 22.4%. Oral ulcers were documented in 18.3% of patients, while seizures were uncommon (6.8%). Anemia was found in 59.7% of the cohort, and more than half (56.9%) experienced edema. Dyspnea affected 26.4% of patients, while eye redness was reported in 9.6% (Table 2).

**Table 2:** Clinical manifestations in patients of systemic lupus erythematosus.

Clinical manifestation	Number of cases (%)
Malar rash	25 (32.5%)
Fever	11 (14.9%)
Rash other than malar	18 (24.3%)
Photosensitivity	10 (12.8%)
Serositis	17 (22.4%)
Oral ulcers	13 (18.3%)
Non-erosive arthritis	47 (63.5%)
Seizures	5 (6.8%)
Anemia	43 (59.7%)
Edema	41 (56.9%)
Eye redness	7 (9.6%)
Dyspnea	19 (26.4%)

Laboratory findings showed widespread proteinuria, with nearly half of the patients having nephrotic-range proteinuria. Hematuria and hypoalbuminemia were also common. High levels of serum creatinine and BUN were noted in one-third and nearly half of the patients, respectively. Anemia and elevated inflammatory markers were observed in most cases, and the majority were ANA-positive, with three-quarters also testing positive for anti-dsDNA antibodies. Corticosteroids and other immunosuppressants were frequently used as part of treatment (Table 3).

**Table 3:** Laboratory findings and treatment modalities in patients with lupus nephritis DMARDs: disease-modifying antirheumatic drugs.

Variable	Frequency, n (%)
High creatinine (serum creatinine above 1.5 mg /dl)	24 (33.3%)
High blood urea nitrogen (BUN) (BUN level above 20 mg/dl)	34 (47.9%)
Low C3 complement (C3 level below 60 mg/dl)	38 (61.3%)
Low C4 complement (C4 level below 15 mg/dl)	36 (62.1%)
Hypoalbuminemia (serum albumin below 3 gm/dl)	30 (46.2%)
Anemia (hemoglobin level below 13 gm/dl)	60 (80%)
Low platelets below 150,000 platelets per mcL	13 (17.3%)
High C-reactive protein (above 5 mg/L)	46 (86.8%)
High erythrocyte sedimentation rate (above 15 mm/hr)	43 (86%)
Proteinuria	69 (90.8%)
Nephrotic range proteinuria: >3.5 gm/d	37 (48.7%)
Hematuria	54 (70.1%)
Antinuclear antibody (+)	59 (92.2%)
Anti-double-stranded DNA (+)	45 (75.0%)
Steroid use	66 (85.7%)
Drugs (disease-modifying antirheumatic drugs)	48 (66.7%)
		Drugs (immunosuppressive other than steroids and DMARDs)	53 (71.6%)

Most patients remained under follow-up with stable disease. A minority developed ESRD, and two patients died due to LN complications. Male gender, hypertension, and elevated serum creatinine and BUN levels were significantly associated with poorer outcomes, particularly progression to dialysis. In contrast, variables such as age, diabetes, SLE duration, nephrotic-range proteinuria, low complement levels, and autoantibody status did not show statistically significant correlations with mortality or dialysis. Histological class of LN also did not significantly predict adverse outcomes (Table 4).

**Table 4:** Association between characteristics and outcomes. P-values were calculated using Fisher’s exact test.

Characteristics	Follow-up	On dialysis	Death	P-value (Fisher’s exact test)
Age group (years), No. (%)				
1-14	2 (3.2%)	0 (0%)	0 (0%)	0.249
15-29	28 (44.5%)	4 (80%)	2 (100%)	
30-45	27 (42.9%)	0 (0%)	0 (0%)	
>45	6 (9.5%)	1 (20%)	0 (0%)	
Gender, No. (%)				
Female	53 (84.1%)	0 (0%)	2 (100%)	<0.001
Male	10 (15.9%)	5 (100%)	0 (0%)	
Diabetes mellitus, No. (%)	5 (7.9%)	1 (20%)	0 (0%)	0.482
Hypertension, No. (%)	17 (27%)	4 (80%)	0 (0%)	0.035
Duration of systemic lupus erythematosus, No. (%)				
<1 year	4 (7.3%)	1 (20%)	0 (0%)	0.687
1-4 years	19 (34.5%)	2 (40%)	1 (100%)	
5-10 years	24 (43.6%)	2 (40%)	0 (0%)	
>10 years	8 (14.5%)	0 (0%)	0 (0%)	
Nephrotic range proteinuria: >3.5 gm/d	30 (48.4%)	3 (60%)	2 (100%)	0.59
Hematuria, No. (%)	48 (76.2)	2 (40%)	2 (100%)	0.161
Low platelets below 150,000 platelets per mcL	9 (14.3%)	1 (20%)	2 (100%)	0.059
High creatinine (serum creatinine above 1.5 mg/dl)	15 (24.6%)	5 (100)	2 (100%)	<0.001
High blood urea nitrogen (BUN): (BUN level above 20 mg/dl)	17 (31.5%)	4 (100%)	2 (100%)	0.01
Low C3 complement (C3 level below 60 mg/dl )	25 (48.1%)	5 (100%)	2 (100%)	0.127
Anti-double-stranded DNA (+), No. (%)	37 (74%)	4 (100)	1 (100)	0.717
Anti-nucleotide antibody (+), No. (%)	47 (90.4%)	5 (100%)	1 (100%)	1
Light microscopy fibrosis, No. (%)	25 (39.7)	4 (80)	2 (100)	0.201
Lupus nephritis class, No. (%)				
Class II	5 (8.9%)	0 (0%)	0 (0%)	0.247
Class III	13 (23.2%)	1 (25%)	0 (0%)	
Class IV	26 (46.4%)	2 (50%)	2 (100%)	
Class V	10 (17.9%)	0 (0%)	0 (0%)	
Class VI	0 (0%)	1 (25%)	0 (0%)	
Class IV+V	2 (3.6%)	0 (0%)	0 (0%)	

Further analysis showed class IV LN was most frequent among younger patients. Female patients predominated across all LN classes, except for class VI. BUN levels and the presence of nephrotic-range proteinuria varied significantly with LN class, suggesting their potential role in identifying severe histologic patterns. Other laboratory variables did not differ significantly by class (Table 5).

**Table 5:** The association between lupus nephritis class and general characteristics and lab results. P-values were calculated using Fisher’s exact test.

Variable	Class I	Class II	Class III	Class IV	Class V	Class VI	P-value (Fisher’s exact test)
Age group (years), No. (%)							
1-14	0 (0%)	0 (0%)	0 (0%)	1 (3%)	1 (8.3%)	0 (0%)	0.011
15-29	0 (0%)	1 (16.7%)	8 (50%)	21 (63.6%)	3 (25%)	1 (100%)	
30-45	0 (0%)	3 (50%)	6 (37.5%)	9 (27.3%)	8 (66.7%)	0 (0%)	
>45	2 (100%)	2 (33.3%)	2 (12.5%)	2 (6.1%)	0 (0%)	0 (0%)	
Gender, No. (%)							
Female	2 (100%)	5 (83%)	11 (68.8%)	26 (78.8%)	12 (100%)	0 (0%)	0.121
Male	0 (0%)	1 (16.7%)	5 (31.3%)	7 (21.2%)	0 (0%)	1 (100%)	
Duration of systemic lupus erythematosus, No. (%)							
<1 year	0 (0%)	0 (0%)	0 (0%)	4 (14.8%)	0 (0%)	0 (0%)	0.368
1-4 years	0 (0%)	2 (40%)	2 (14.3%)	12 (44.4%)	5 (45.5%)	0 (0%)	
5-10 years	1 (100%)	3 (60%)	8 (57.1%)	9 (33.3%)	5 (45.5%)	1 (100%)	
>10 years	0 (0%)	0 (0%)	4 (28.6%)	2 (7.4%)	1 (9.1%)	0 (0%)	
High creatinine (serum creatinine above 1.5 mg/dl)	1 (100%)	0 (0%)	4 (30.8%)	13 (48.1%)	2 (20%)	0 (0%)	0.97
Low C3 complement (C3 level below 60 mg/dl)	1 (50%)	0 (0%)	7 (50%)	18 (66.7%)	7 (77.8%)	0 (0%)	0.107
Low C4 complement, No. (%)	2 (100%)	1 (25%)	5 (38.5%)	19 (76%)	4 (50%)	0 (0%)	0.087
High blood urea nitrogen (BUN): (BUN level above 20 mg/dl)	1 (100%)	0 (0%)	4 (28.6%)	16 (59.3%)	2 (22.2%)	0 (0%)	0.013
Nephrotic range proteinuria: >3.5 gm/d	0 (0%)	0 (0%)	6 (37.5%)	20 (62.5%)	6 (50%)	1 (100%)	0.034
Hematuria, No. (%)	0 (0%)	4 (80%)	10 (62.5%)	25 (75.8%)	8 (66.7%)	1 (100%)	0.327

## Discussion

This is the first retrospective study from Palestine to characterize the clinical spectrum, histopathological classifications, treatment patterns, and outcomes of patients with biopsy-proven LN. The research identifies factors that guide future treatment strategies for patients with SLE who have renal involvement, as well as critical prognostic indicators associated with poor renal outcomes and mortality.

Demographics and clinical presentation

In this study, a predominance of females (80.8%) was found in the patient population, consistent with the known higher prevalence of SLE in women, as reported in previous studies [10,12-14]. The peak age of onset was between 15 and 29 years, which closely resembled findings from other studies conducted in different countries [10,13].

The average interval between SLE diagnosis and LN confirmation was 5.5 ± 4.1 years, which is longer than previously reported [12,13]. Potentially indicating delays in referral, diagnosis, or access to biopsy services in the Palestinian healthcare system. These delays may contribute to more advanced disease at the time of presentation.

Consistent with other regional reports, non-renal symptoms such as arthritis (63.5%) and malar rash (32.5%) were the most common symptoms [10,12,13]. However, lower rates of photosensitivity and mucosal involvement compared to Saudi and Indian cohorts might be the result of environmental or ethnic variability.

Laboratory and immunological findings

Laboratory results consistent with established immunologic markers of LN [12,13], including their use in diagnosis and monitoring, included hypocomplementemia (low C3 in 61.3% and low C4 in 62.1%), ANA positivity (92.2%), and anti-dsDNA positivity (75%).

A major predictor of outcome was renal function at the time of the biopsy. High serum creatinine (>1.5 mg/dL) and BUN (>20 mg/dL) were strongly associated with ESRD and mortality (p < 0.001 and p = 0.01, respectively). In resource-limited settings, such as Palestine, the high predictive value of BUN, which is less commonly documented in the literature, justifies additional research as a valuable prognostic marker.

More than 90% of patients had proteinuria, and nearly half (48.7%) of them had nephrotic-range proteinuria (>3.5 g/day). As in previous studies, nephrotic-range proteinuria was significantly associated with higher LN class, particularly class IV (p = 0.034), reaffirming its value as a non-invasive marker of disease severity [12,13].

Hematuria was prevalent in 70% of patients, higher than rates reported in Saudi Arabia (43.1%) and India (59.1%) [12,13]. Anemia (80%) and thrombocytopenia (17.3%) were similar to rates in Saudi Arabia and India, although anemia was significantly higher compared to the United States (45%) [12-14].

Histological patterns

According to histopathology, the distribution of LN classes reflects worldwide patterns, with class IV (47.1%) being the most common, followed by class III (22.9%) and class V (14.3%) [12,13]. We found a significant association between age group and LN class (p = 0.011), with all class I patients being over 45 years old. Half of the patients with class III and the majority of those with class IV (63.6%) were in the 15-29 years age group. However, no significant associations were found between LN class and mortality or dialysis outcomes, possibly due to the small number of adverse outcomes or limitations in biopsy detail due to the lack of electron microscopy in Palestine.

Gender, hypertension, and outcomes

The results showed significant gender differences: all dialysis patients were male, and all recorded deaths were of females (p < 0.001). Male gender may be linked to worse renal outcomes, according to some research [15], but other studies have not consistently found a link [16]. Future prospective studies should investigate this gender-based difference in results in more detail.

Hypertension (HTN) was observed in 30.7% of patients, which is lower than the rates in Saudi Arabia (45.8%) and Jordan (74.7%) [10,13]. Nevertheless, HTN showed a strong association with dialysis dependency and adverse outcomes (p = 0.035), supporting prior findings that highlight the role of hypertension as a modifiable risk factor that contributes to LN progression [13,14]. Our data also suggest that HTN, particularly when coexisting with elevated creatinine or BUN, significantly worsens prognosis, which emphasizes the significance of strict blood pressure management for LN patients.

Treatment patterns and implications

Treatment approaches followed standard LN protocols; 85.7% of patients received corticosteroids, 66.7% received DMARDs, and 71.6% received additional immunosuppressive medications. Two patients (2.6%) passed away as a result of LN complications, and five patients (6.4%) advanced to ESRD and needed dialysis. These rates are marginally lower than those found in Saudi Arabia (9% dialysis, 6% mortality) and Lebanon (11% dialysis, 13% mortality); however, this discrepancy may be partially explained by variations in sample size and follow-up period [13,17].

Strengths and limitations

The main strengths of this study are its novelty as the first multi-center retrospective analysis of LN in Palestine, its relatively large cohort size for the region, and the integration of clinical, laboratory, and histopathological data. This study has several inherent limitations. Since it is a retrospective design, it cannot prove causation and is prone to documentation bias. The evaluation of activity and chronicity indices, although primarily based on LM reports, could not be fully performed, and the complete classification of LN was not possible due to the lack of electron microscopy. Furthermore, measurement irregularities may have been caused by inter-center variations in laboratory procedures.

## Conclusions

This study provides the first comprehensive assessment of LN in a Palestinian cohort. The findings underscore a high prevalence of severe histologic forms such as class IV, with significant associations between poor outcomes and clinical indicators like male gender, hypertension, and impaired renal function. In our cohort, a subset of patients progressed to ESRD or died despite treatment adherence. These findings underscore the poor outcomes observed in some patients and suggest that factors such as hypertension and elevated creatinine levels may identify those at higher risk. The study also indicates that BUN, often overlooked, may be a useful prognostic marker in resource-limited settings. These findings support the need for better diagnostic tools, consistent follow-up, and gender-sensitive interventions in managing LN. Future longitudinal studies are recommended to further validate these observations and inform evidence-based practice.
